# *N*,*N*′,*N*′′ *versus N*,*N*′,*O* imine-containing coordination motifs: ligand-directed synthesis of mononuclear and binuclear Cu^II^ compounds

**DOI:** 10.1107/S2056989017013652

**Published:** 2017-09-29

**Authors:** Raphael Enoque Ferraz de Paiva, Douglas Hideki Nakahata, Marcos Alberto de Carvalho, Fernando Rodrigues Goulart Bergamini, Pedro Paulo Corbi

**Affiliations:** a Institute of Chemistry, University of Campinas - UNICAMP, Campinas – SP 13083-970, Brazil

**Keywords:** crystal structure, mononuclear copper(II) complexes, binuclear copper(II) complexes, imine, tridentate ligand

## Abstract

It is demonstrated here that tridentate imine ligands can control the nuclearity of copper(II) complexes based on the donor atoms present in the ligand. The *N*,*N*′,*N*′′-donating imine ligand led to a mononuclear compound, namely di­chlorido­[*N*,*N*-dimethyl-*N*′-(pyridin-2-yl­methyl­idene)ethane-1,2-di­amine]copper(II) monohydrate, [CuCl_2_(C_10_H_15_N_3_)]·H_2_O, **1**, while the *N*,*N*′,*O*-donating imine ligand produced a binuclear metal complex, namely μ_2_-chlorido-di­chlorido­(μ_2_-2-{[2-(di­methyl­amino)­ethyl]imino­methyl}phenolato)(*N*,*N*-dimethyl­ethylene­di­amine)­dicopper(II) 0.11-hydrate, [Cu_2_(C_11_H_15_N_2_O)Cl_3_(C_4_H_12_N_2_)]·0.11H_2_O, **2**. The structure of **2** is a remarkable example of a binuclear copper(II) complex containing a single substituted 2-imino­methyl­phenolate ligand that has two copper(II) sites in square-pyramidal coordination.

## Abstract 2

It is shown that tridentate imine ligands can control the nuclearity of copper(II) complexes based on the donor atoms present in the ligand. While the *N*,*N*′,*N*′′-donating imine ligand led to a mononuclear compound, the *N*,*N*′,*O*-donating imine ligand produced a binuclear metal complex.

## Chemical context   

Copper(II) complexes with imine ligands have attracted much attention in the past few decades due to a variety of possible applications, including catalysis [aerobic oxidation of alcohols (Nairn *et al.*, 2006 ▸; Alaji *et al.*, 2014 ▸), olefin epoxidation (Das *et al.*, 1997 ▸) and ring-opening reactions (John *et al.*, 2007 ▸)], and also in medicinal chemistry for both anti­bacterial (Ali *et al.*, 2015 ▸) and anti­tumour applications (Creaven *et al.*, 2010 ▸; Pervez *et al.*, 2016 ▸).
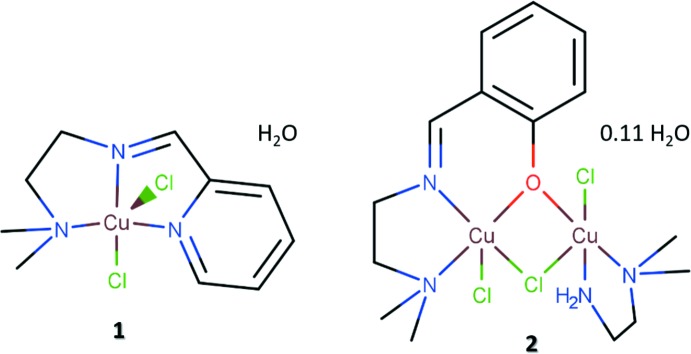


Nonmacrocyclic binuclear copper compounds are of inter­est because they can serve as models for metalloproteins and metalloenzymes, as well as representing inter­esting subjects for studying mol­ecular magnetism. Strong magnetic exchange is present in the two copper(II) sites of haemocyanin (Chen & Solomon, 2004 ▸), which represents a challenge that must be considered when synthetic models are developed. One strategy, introduced by Robson (1970 ▸), makes use of symmetrical imino ligands containing a phenolate bridge to keep the Cu^II^ atoms close in space. Imines represent an inter­esting class of ligands because they can be easily synthesized and fine-tuned to the desired application by introducing extra donor atoms or groups with the desired steric properties into the side chains. A limited number of binuclear copper(II) compounds containing substituted 2-imino­methyl­phenole ligands have been reported in the literature (Gao *et al.*, 2011 ▸; Tang *et al.*, 2008 ▸). This kind of structure, where the polydentate ligand has fewer donor atoms than the coordination number of the metal centre, is of inter­est for the design of more flexible binuclear model compounds.

We describe here the crystal structures of mononuclear (**1**) and binuclear (**2**) copper(II) complexes with tridentate imine-containing ligands obtained by a one-pot synthetic method. The nuclearity of the complexes was shown to be directed by the different donor atoms present in the imine ligand.

## Structural commentary   

**Figure 1:**
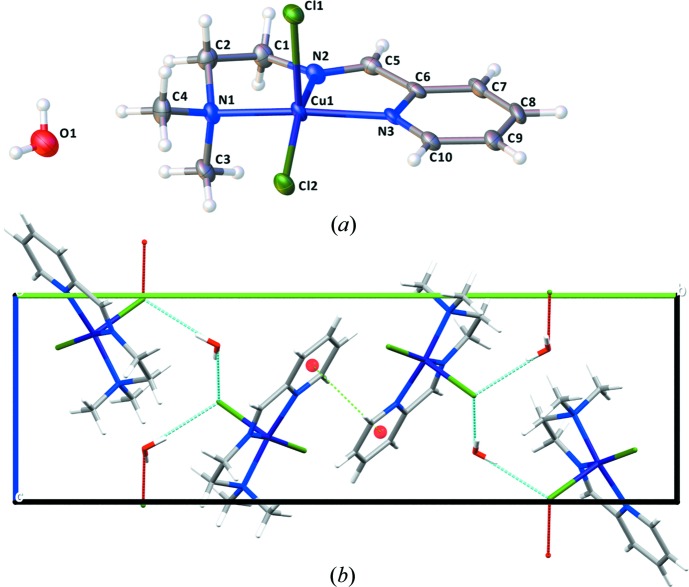
(*a*) Mol­ecular view of the structure of **1**. Displacement ellipsoids are drawn at the 50% probability level. H atoms are not labelled for clarity. (*b*) Packing of the crystal structure of **1**, viewed along the *a* axis, highlighting the hydrogen-bonded chain (comprising the water mol­ecules and the axial chloride ligand) as well as the π-stacking.

The mononuclear compound **1** has the central Cu^II^ cation in a square-pyramidal coordination environment (Fig. 1 ▸ *a*). The Cu^II^ cation is displaced from the least-squares plane defined by the four coordinating atoms of the square base (N1, N2, N3 and Cl2) by 0.334 Å. The bond lengths to these atoms are: Cu—N1 = 2.060 (2), Cu—N2 = 1.978 (2), Cu—N3 = 2.058 (2) and Cu—Cl2 = 2.2639 (8) Å; the Cu—Cl bond length to the apical Cl1 atom that completes the first coordination sphere is considerably longer, at 2.5013 (8) Å. In order to assess the coordination geometry of copper(II) more qu­anti­tatively, the τ_5_ index as defined by Addison *et al.* (1984 ▸) can be used. A perfect square-pyramidal coordination geometry is defined by τ_5_ = 0.0, while it is 1.0 for a perfect trigonal–bipyramidal coordination geometry. For compound **1**, τ_5_ is 0.059, indicating an almost perfect square-pyramidal coordination geometry.

**Figure 2:**
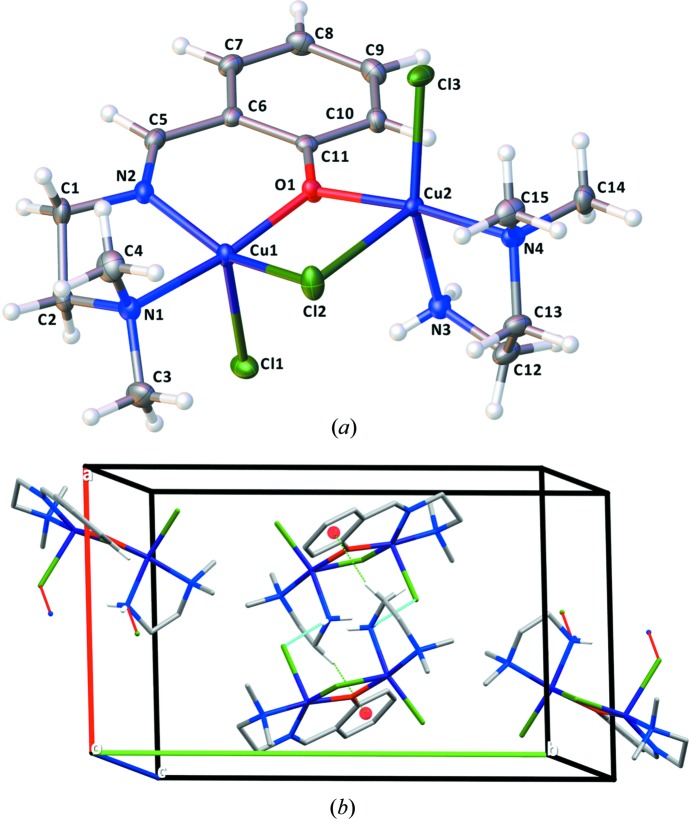
(*a*) Mol­ecular structure of compound **2**. Displacement ellipsoids are drawn at the 50% probability level. H atoms are not labelled for clarity. (*b*) Packing of the crystal structure of **2**, highlighting the network of hydrogen bonds between the terminal amine group of the ligand and the chloride ligands as well as the C—H⋯π inter­actions. H atoms not participating in hydrogen bonding have been omitted for clarity.

The binuclear compound **2** has two copper(II) cations, both in a square-pyramidal coordination environment (Fig. 2 ▸ *a*). The presence of the phenolate group in the structure of the imine ligand directs the reaction with copper(II) cations to form a binuclear coordination compound, in contrast with the mononuclear species **1** obtained when a pyridine group is present in the ligand. Atoms Cu1 and Cu2 in **2** are displaced from the least-squares plane defined by the four coordinating atoms of the square base (N1, N2, O1 and Cl2 for Cu1; N3, N4, O1 and Cl3 for Cu2) by 0.299 and 0.170 Å, respectively. The distances from the central copper(II) cations to these ligating atoms are: Cu1—N1 = 2.068 (2), Cu1—N2 = 1.959 (2), Cu1—O1 = 1.968 (1) and Cu1—Cl2 = 2.2958 (5) Å; Cu2—N3 = 2.021 (2), Cu2—N4 = 2.040 (2), Cu2—Cl3 = 2.2501 (5) and Cu2—O1 = 2.004 (1) Å. The two Cu—Cl distances to the apical Cl atoms are likewise longer, Cu1—Cl1 = 2.5476 (5) Å and Cu2—Cl2 = 2.5938 (5) Å. The Cu⋯Cu distance within the binuclear complex is 3.2525 (5) Å. In compound **2**, the τ_5_ index for Cu1 is 0.294 and for Cu2 0.260, indicating more distorted square-pyramidal coordination environments for both central copper(II) cations.

After refining the structure of the binuclear compound **2**, a solvent-accessible void of 42 Å^3^ was detected by a *PLATON* analysis (Spek, 2009 ▸). The highest residual electron-density peak fitted perfectly within this void. We have modelled the corresponding site as an O atom of a partially occupied water mol­ecule, showing an occupancy of 0.11. Given the low occupancy, this water mol­ecule is not represented in the mol­ecular view nor in the crystal packing (Fig. 2 ▸).

## Supra­molecular features   

The presence of a water mol­ecule in the crystal structure of the mononuclear compound **1** leads to the formation of a hydrogen-bonded chain along [101] involving the apical ligand Cl1 (Fig. 1 ▸ *b* and Table 1 ▸). In addition, a short contact between the C—H group of the imine group and the apical Cl1 ligand is observed (C5—H5⋯Cl1, Table 1 ▸). Finally, a similar C—H⋯Cl inter­action between an aromatic H atom of the pyridine ring and the Cl2 ligand of the square base likewise contributes to the packing in the solid state (C9—H9⋯Cl2, Table 1 ▸). Besides these hydrogen bonds, an offset π–π stacking is observed between adjacent pyridine rings [centroid-to-centroid distance of 3.5709 (18) Å; symmetry code: −*x*, −*y*, −*z*].

In terms of inter­molecular contacts, a single set of hydrogen bonds is present in the crystal structure of **2**, established between the non-substituted terminal amine group of *N*,*N*-di­methyl­ethylenedi­amine and the apical chloride ligand Cl1 (Fig. 2 ▸ *b* and Table 2 ▸). Similar to compound **1**, a nonclassical hydrogen bond between an aromatic H atom of the phenolic ring and the Cl2 ligand also contributes to the inter­molecular network (C8—H8⋯Cl2, Table 2 ▸). Differing from the structure of **1**, a C—H⋯π inter­action is observed for compound **2**, with a C12—H12⋯centroid(phen­yl) distance of 3.393 (2) Å (symmetry code: −*x* + 1, −*y* + 1, −*z* + 1). The partly occupied water mol­ecule participates in a hydrogen bond with the μ_2_-bridging Cl2 ligand (Table 2 ▸).

## Database survey   

**Figure 3:**
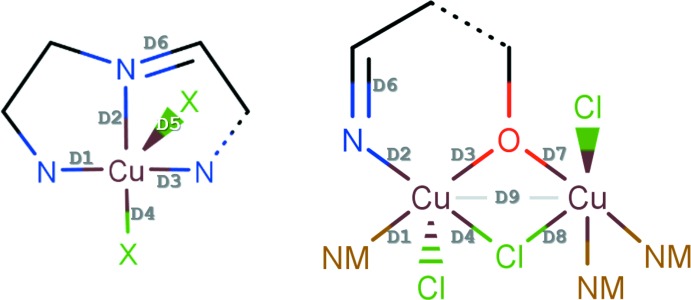
Structures used as queries for the search of the CSD. Analogues of both the mononuclear and binuclear Cu^II^ compounds were searched for. NM represents any non-metal, dashed lines represent any bond type and D9 represents the Cu⋯Cu distance.

The structures of the mononuclear and binuclear copper(II) compounds **1** and **2** were compared with analogues found in the Cambridge Structural Database (CSD; Groom *et al.*, 2016 ▸), using the queries shown in Fig. 3 ▸. Only binuclear Cu^II^ compounds containing a single μ_2_-(mono­imino­methyl)­phenolate ligand were considered as analogues of **2**. A total of 12 hits were found as analogues of **1**, while 11 hits were found for analogues of **2**, including both mono- and bis­(imino­methyl)­phenolate ligands. Averages of selected bond lengths (see representations in Fig. 3 ▸) were obtained using *ConQuest* (Version 1.19) and the statistical analysis module in *Mercury* (Version 3.9) (Macrae *et al.*, 2008 ▸). The averaged values are collated in Table 3 ▸ and are in good agreement with the bond lengths in the structures of **1** and **2**.

**Figure 4:**
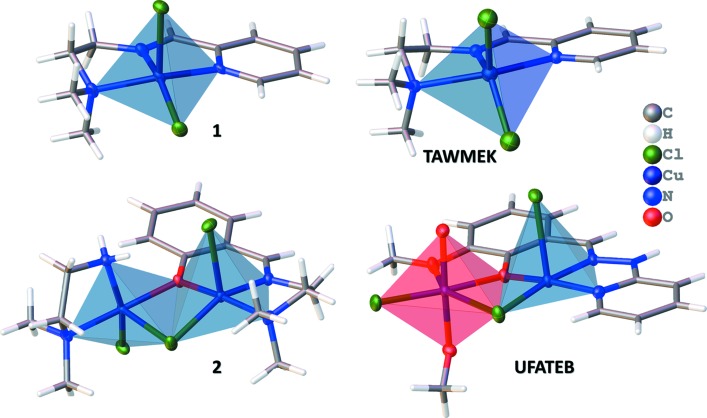
Polyhedral representation of the coordination spheres of Cu^II^ in **1** and **2**, compared with analogous compounds previously reported in the literature. Square-pyramidal coordination spheres (typical and distorted) are represented in blue and the octa­hedral coordination sphere in red.

The closest relation to **1** is associated with the nonhydrated analogue (CCDC entry TAWMEK; Yuan & Zhang, 2005 ▸), which has the Cu^II^ cation in a more distorted square-pyramidal coordination geometry than **1**, with the following bond lengths: Cu—N1 = 2.275 (2), Cu—N2 = 2.104 (2) and Cu—N3 = 2.236 (2) Å, and almost identical Cu—Cl1 = 2.2573 (5) and Cu—Cl2 = 2.22561 (6) Å distances. The Cu^II^ cation is displaced from the mean plane defined by the four coordinating atoms of the square base by 0.622 Å. While for **1** τ_5_ = 0.0593, for the structure of TAWMEK τ_5_ = 0.302. The differences in the coordination environment of copper(II) probably arise as a consequence of the presence of the hydrogen-bonded network established between the chloride ligands and the water mol­ecules in the crystal structure of **1**. The coordination spheres around the Cu^II^ cations in **1** and TAWMEK are compared in Fig. 4 ▸.

Regarding the binuclear compound **2**, the search returned only two examples of binuclear Cu^II^ complexes containing a single μ_2_-(mono­imino­methyl)­phenolate ligand [VAMJIE (Gao *et al.*, 2011 ▸) and UFATEB (Tang *et al.*, 2008 ▸)]. The two structures have one Cu^II^ cation in a square-pyramidal environment, comprising the tridentate imine ligand, and one octa­hedrally surrounded Cu^II^ site, bridged by the phenolate and a chloride ligand. Structure **2**, on the other hand, comprises a binuclear copper(II) complex with a single μ_2_-(mono­imino­methyl)­phenolate ligand that has two Cu^II^ co­ordination sites in square-pyramidal environments. The co­ordination spheres around the two Cu^II^ cations in **2** and UFATEB are compared in Fig. 4 ▸.

## Synthesis and crystallization   

Copper(II) chloride dihydrate was purchased from Vetec (Brazil). *N*,*N*-di­methyl­ethylene­di­amine, pyridine-2-carbox­aldehyde and salicyl­aldehyde were purchased from Sigma–Aldrich and used without further purification.

Compound **1**, C_10_H_15_Cl_2_CuN_3_·H_2_O, was obtained as follows. In a 10 ml beaker, *N*,*N*-di­methyl­ethylene­di­amine (0.10 mmol, 65.6 µl) was combined with pyridine-2-carbox­aldehyde (0.10 mmol, 10 µl) in methanol (200 µl). The reaction was carried out at room temperature for 24 h. Afterwards, solid CuCl_2_·2H_2_O (0.10 mmol, 2.8 mg) was added to the reaction mixture. A polycrystalline green compound was obtained, filtered off and washed with small amounts of cold methanol. Elemental analysis was performed on a Perkin–Elmer CHNS-O 2400. Analysis, calculated for C_10_H_15_Cl_2_CuN_3_·H_2_O: C 36.4, H 5.2, N 12.7%; found: C 36.8, H 4.8, N 13.0%. The supernatant was transferred to an amber flask and green crystals suitable for single-crystal X-ray diffraction were obtained by slow evaporation.

Compound **2** (C_15_H_27_Cl_3_Cu_2_N_4_O·0.11H_2_O) was obtained following the same synthetic procedure as used for **1**, but replacing pyridine-2-carbox­aldehyde by salicyl­aldehyde (11 µl). Green needle-like crystals of **2** were obtained by slow evaporation of the supernatant. Since only a few crystals were obtained, no further analytical data were acquired.

## Refinement details   

Crystal data, data collection and structure refinement details are summarized in Table 4 ▸. H atoms were placed in calculated positions, with C—H = 0.99 (CH_2_) or 0.95 Å (CH), with *U*_iso_(H) = 1.2*U*_eq_(C), and C—H = 0.98 Å (CH_3_) and *U*_iso_(H) = 1.5*U*_eq_(C). For structure **1**, the H atoms of the water mol­ecule were refined with an O—H distance restraint of 0.82 (1) Å and a H⋯H separation of 1.29 (2) Å, and with *U*_iso_(H) = 1.5*U*_eq_(O). For structure **2**, the H atoms of the amine functionality (H3*A* and H3*B*) were refined freely. The occupancy of the partly occupied water solvent mol­ecule was refined to a value of 0.11 (1); for this mol­ecule, H atoms were not located and they were not considered in the final model.

## Supplementary Material

Crystal structure: contains datablock(s) global, 2, 1. DOI: 10.1107/S2056989017013652/wm5417sup1.cif

Structure factors: contains datablock(s) 1. DOI: 10.1107/S2056989017013652/wm54171sup2.hkl

Structure factors: contains datablock(s) 2. DOI: 10.1107/S2056989017013652/wm54172sup3.hkl

CCDC references: 1576100, 1576099

Additional supporting information:  crystallographic information; 3D view; checkCIF report

## Figures and Tables

**Table 1:** Hydrogen-bond geometry (Å, °) for **1** [Chem scheme1]

*D*—H⋯*A*	*D*—H	H⋯*A*	*D*⋯*A*	*D*—H⋯*A*
O1—H1*D*⋯Cl1^i^	0.84 (1)	2.45 (1)	3.282 (3)	173 (5)
O1—H1*C*⋯Cl1^ii^	0.84 (1)	2.43 (1)	3.252 (3)	166 (4)
C5—H5⋯Cl1^iii^	0.95	2.73	3.669 (3)	171
C9—H9⋯Cl2^iv^	0.95	2.86	3.543 (3)	130

Symmetry codes: (i) 
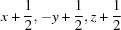
; (ii) 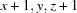; (iii) 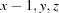; (iv) 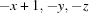.

**Table 2:** Hydrogen-bond geometry (Å, °) for **2** [Chem scheme1]

*D*—H⋯*A*	*D*—H	H⋯*A*	*D*⋯*A*	*D*—H⋯*A*
N3—H3*A*⋯Cl1^i^	0.87 (2)	2.46 (2)	3.2543 (17)	153 (2)
N3—H3*B*⋯Cl1	0.84 (2)	2.74 (2)	3.5207 (1)	154 (2)
C8—H8⋯Cl2^ii^	0.95	2.81	3.6841 (19)	153
O1*W*⋯Cl2^i^			3.17 (2)	

Symmetry codes: (i) 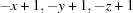; (ii) 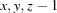.

**Table 3:** Averages of selected bond lengths (as represented in Fig. 3 ▸) obtained by searching the CSD for compounds analogous to **1** and **2**

	**1**	Analogues of **1**, average of 12 hits	**2**	Analogues of **2**, average of 11 hits
D1	2.060 (2)	2.06 (7)	2.067 (2)	2.01 (4)
D2	1.978 (2)	1.99 (4)	1.959 (2)	1.98 (3)
D3	2.058 (2)	2.06 (6)	1.968 (1)	1.969 (19)
D4	2.2639 (8)	2.240 (11)	2.5477 (5)	2.28 (3)^sb^, 2.60 (5)^ap^
D5	2.5014 (9)	2.487 (17)		
D6	1.273 (3)	1.269 (15)	1.278 (3)	1.281 (6)
D7			2.004 (1)	2.01 (3)
D8			2.5939 (5)	2.287 (19)^sb^, 2.74 (12)^ap^
D9			3.2525 (5)	3.24 (10)

Notes: sb = ligands at the square base of the polyhedron; ap = ligands at the apical position.

**Table 4:** Experimental details

	**1**	**2**
Crystal data		
Chemical formula	[CuCl_2_(C_10_H_15_N_3_)]·H_2_O	[Cu_2_(C_11_H_15_N_2_O)Cl_3_(C_4_H_12_N_2_)]·0.11H_2_O
*M*_r_	329.70	514.77
Crystal system, space group	Monoclinic, *P*2_1_/*n*	Monoclinic, *P*2_1_/*c*
Temperature (K)	150	150
*a*, *b*, *c* (Å)	6.9667 (5), 24.735 (2), 7.9294 (6)	11.0838 (4), 18.0949 (7), 10.6610 (4)
β (°)	103.693 (4)	101.474 (2)
*V* (Å^3^)	1327.55 (18)	2095.44 (14)
*Z*	4	4
Radiation type	Cu *K*α	Cu *K*α
μ (mm^−1^)	5.93	6.12
Crystal size (mm)	0.27 × 0.05 × 0.05	0.08 × 0.06 × 0.04

Data collection		
Diffractometer	Bruker *APEX* CCD area-detector	Bruker APEXII CCD area-detector
Absorption correction	Multi-scan (*SADABS*; Bruker, 2010 ▸)	Multi-scan (*SADABS*; Bruker, 2010 ▸)
*T*_min_, *T*_max_	0.522, 0.753	0.654, 0.753
No. of measured, independent and observed [*I* > 2σ(*I*)] reflections	7065, 2361, 2199	11774, 3680, 3333
*R*_int_	0.041	0.024
(sin θ/λ)_max_ (Å^−1^)	0.603	0.602

Refinement		
*R*[*F* ^2^ > 2σ(*F* ^2^)], *wR*(*F* ^2^), *S*	0.040, 0.108, 1.10	0.022, 0.056, 1.03
No. of reflections	2361	3680
No. of parameters	162	248
No. of restraints	3	0
H-atom treatment	H atoms treated by a mixture of independent and constrained refinement	H atoms treated by a mixture of independent and constrained refinement
Δρ_max_, Δρ_min_ (e Å^−3^)	0.65, −0.60	0.32, −0.28

Computer programs: *APEX2* (Bruker, 2010 ▸), *SAINT* (Bruker, 2010 ▸), *SHELXS97* (Sheldrick, 2008 ▸), *SHELXL2014* (Sheldrick, 2015 ▸), *OLEX2* (Dolomanov *et al.*, 2009 ▸), *Mercury* (Macrae *et al.*, 2008 ▸) and *publCIF* (Westrip, 2010 ▸).

## supplementary crystallographic information

### Dichlorido[N,N-dimethyl-N'-(pyridin-2-ylmethylidene)ethane-1,2-diamine]copper(II) monohydrate (1) Crystal data

**Table:**

[CuCl_2_(C_10_H_15_N_3_)]·H_2_O	*F*(000) = 676
*M**_r_* = 329.70	*D*_x_ = 1.650 Mg m^−^^3^
Monoclinic, *P*2_1_/*n*	Cu *K*α radiation, λ = 1.54178 Å
*a* = 6.9667 (5) Å	Cell parameters from 5700 reflections
*b* = 24.735 (2) Å	θ = 6.0–68.2°
*c* = 7.9294 (6) Å	µ = 5.93 mm^−^^1^
β = 103.693 (4)°	*T* = 150 K
*V* = 1327.55 (18) Å^3^	Needle, green
*Z* = 4	0.27 × 0.05 × 0.05 mm

### Dichlorido[N,N-dimethyl-N'-(pyridin-2-ylmethylidene)ethane-1,2-diamine]copper(II) monohydrate (1) Data collection

**Table:**

Bruker APEX CCD area-detector diffractometer	2199 reflections with *I* > 2σ(*I*)
Detector resolution: 8.3333 pixels mm^-1^	*R*_int_ = 0.041
φ and ω scans	θ_max_ = 68.4°, θ_min_ = 6.0°
Absorption correction: multi-scan (SADABS; Bruker, 2010)	*h* = −8→8
*T*_min_ = 0.522, *T*_max_ = 0.753	*k* = −29→29
7065 measured reflections	*l* = −9→5
2361 independent reflections	

### Dichlorido[N,N-dimethyl-N'-(pyridin-2-ylmethylidene)ethane-1,2-diamine]copper(II) monohydrate (1) Refinement

**Table:**

Refinement on *F*^2^	3 restraints
Least-squares matrix: full	Hydrogen site location: mixed
*R*[*F*^2^ > 2σ(*F*^2^)] = 0.040	H atoms treated by a mixture of independent and constrained refinement
*wR*(*F*^2^) = 0.108	*w* = 1/[σ^2^(*F*_o_^2^) + (0.0521*P*)^2^ + 1.8713*P*] where *P* = (*F*_o_^2^ + 2*F*_c_^2^)/3
*S* = 1.10	(Δ/σ)_max_ < 0.001
2361 reflections	Δρ_max_ = 0.65 e Å^−^^3^
162 parameters	Δρ_min_ = −0.60 e Å^−^^3^

### Dichlorido[N,N-dimethyl-N'-(pyridin-2-ylmethylidene)ethane-1,2-diamine]copper(II) monohydrate (1) Special details

**Table:**

Geometry. All esds (except the esd in the dihedral angle between two l.s. planes) are estimated using the full covariance matrix. The cell esds are taken into account individually in the estimation of esds in distances, angles and torsion angles; correlations between esds in cell parameters are only used when they are defined by crystal symmetry. An approximate (isotropic) treatment of cell esds is used for estimating esds involving l.s. planes.

### Dichlorido[N,N-dimethyl-N'-(pyridin-2-ylmethylidene)ethane-1,2-diamine]copper(II) monohydrate (1) Fractional atomic coordinates and isotropic or equivalent isotropic displacement parameters (Å^2^)

**Table:**

	*x*	*y*	*z*	*U*_iso_*/*U*_eq_	
Cu1	0.25867 (6)	0.11867 (2)	0.18700 (5)	0.01663 (17)	
Cl1	0.36674 (10)	0.19272 (3)	0.01545 (9)	0.0226 (2)	
Cl2	0.52625 (10)	0.06383 (3)	0.25165 (9)	0.0239 (2)	
N1	0.3071 (3)	0.15604 (10)	0.4256 (3)	0.0196 (5)	
N2	−0.0116 (3)	0.14595 (10)	0.1726 (3)	0.0195 (5)	
N3	0.1025 (3)	0.07572 (10)	−0.0233 (3)	0.0173 (5)	
C1	−0.0402 (4)	0.18436 (13)	0.3060 (4)	0.0244 (7)	
H1A	−0.1083	0.1666	0.3874	0.029*	
H1B	−0.1203	0.2156	0.2518	0.029*	
C2	0.1668 (4)	0.20274 (13)	0.4012 (4)	0.0237 (6)	
H2A	0.2132	0.2314	0.3333	0.028*	
H2B	0.1624	0.2180	0.5157	0.028*	
C3	0.2592 (5)	0.11624 (13)	0.5493 (4)	0.0259 (7)	
H3A	0.1234	0.1034	0.5060	0.039*	
H3B	0.2721	0.1334	0.6628	0.039*	
H3C	0.3506	0.0856	0.5609	0.039*	
C4	0.5104 (5)	0.17632 (14)	0.4964 (4)	0.0289 (7)	
H4A	0.6038	0.1461	0.5099	0.043*	
H4B	0.5184	0.1932	0.6096	0.043*	
H4C	0.5435	0.2030	0.4164	0.043*	
C5	−0.1490 (4)	0.12990 (12)	0.0464 (4)	0.0204 (6)	
H5	−0.2802	0.1433	0.0270	0.024*	
C6	−0.0916 (4)	0.08948 (12)	−0.0667 (4)	0.0195 (6)	
C7	−0.2231 (5)	0.06563 (13)	−0.2042 (4)	0.0240 (7)	
H7	−0.3586	0.0756	−0.2315	0.029*	
C8	−0.1550 (5)	0.02693 (13)	−0.3020 (4)	0.0260 (7)	
H8	−0.2428	0.0103	−0.3979	0.031*	
C9	0.0427 (5)	0.01288 (13)	−0.2578 (4)	0.0240 (6)	
H9	0.0930	−0.0136	−0.3229	0.029*	
C10	0.1664 (4)	0.03815 (12)	−0.1165 (4)	0.0205 (6)	
H10	0.3018	0.0282	−0.0854	0.025*	
O1	0.9383 (4)	0.19474 (11)	0.7408 (4)	0.0429 (7)	
H1D	0.919 (6)	0.2249 (10)	0.692 (6)	0.064*	
H1C	1.055 (3)	0.1978 (17)	0.799 (6)	0.064*	

### Dichlorido[N,N-dimethyl-N'-(pyridin-2-ylmethylidene)ethane-1,2-diamine]copper(II) monohydrate (1) Atomic displacement parameters (Å^2^)

**Table:**

	*U*^11^	*U*^22^	*U*^33^	*U*^12^	*U*^13^	*U*^23^
Cu1	0.0140 (3)	0.0238 (3)	0.0107 (2)	0.00267 (15)	0.00034 (16)	0.00037 (15)
Cl1	0.0228 (4)	0.0262 (4)	0.0186 (3)	−0.0009 (3)	0.0046 (3)	0.0041 (3)
Cl2	0.0182 (4)	0.0300 (4)	0.0210 (4)	0.0068 (3)	−0.0004 (3)	0.0012 (3)
N1	0.0182 (12)	0.0263 (13)	0.0136 (11)	0.0008 (10)	0.0024 (9)	−0.0022 (10)
N2	0.0182 (12)	0.0255 (13)	0.0154 (11)	0.0031 (10)	0.0054 (9)	0.0010 (10)
N3	0.0188 (12)	0.0214 (12)	0.0100 (10)	0.0007 (9)	0.0000 (9)	0.0050 (9)
C1	0.0235 (15)	0.0312 (16)	0.0195 (14)	0.0038 (13)	0.0068 (12)	−0.0030 (13)
C2	0.0253 (16)	0.0256 (16)	0.0213 (14)	0.0018 (12)	0.0076 (12)	−0.0045 (12)
C3	0.0311 (17)	0.0342 (18)	0.0115 (13)	0.0011 (13)	0.0030 (12)	0.0014 (12)
C4	0.0206 (15)	0.0374 (18)	0.0260 (16)	−0.0030 (13)	0.0002 (12)	−0.0091 (14)
C5	0.0158 (14)	0.0247 (15)	0.0202 (14)	0.0013 (11)	0.0033 (11)	0.0063 (12)
C6	0.0202 (14)	0.0234 (15)	0.0130 (12)	0.0005 (11)	0.0004 (11)	0.0083 (11)
C7	0.0208 (15)	0.0310 (17)	0.0163 (13)	−0.0011 (12)	−0.0035 (11)	0.0077 (12)
C8	0.0329 (17)	0.0300 (17)	0.0106 (13)	−0.0071 (13)	−0.0036 (12)	0.0038 (12)
C9	0.0328 (17)	0.0266 (16)	0.0123 (13)	−0.0011 (13)	0.0046 (12)	0.0018 (12)
C10	0.0217 (14)	0.0259 (15)	0.0131 (13)	0.0022 (12)	0.0026 (11)	0.0045 (11)
O1	0.0434 (15)	0.0465 (16)	0.0357 (14)	0.0004 (12)	0.0028 (12)	0.0057 (12)

### Dichlorido[N,N-dimethyl-N'-(pyridin-2-ylmethylidene)ethane-1,2-diamine]copper(II) monohydrate (1) Geometric parameters (Å, º)

**Table:**

Cu1—Cl1	2.5013 (8)	C3—H3B	0.9800
Cu1—Cl2	2.2639 (8)	C3—H3C	0.9800
Cu1—N1	2.060 (2)	C4—H4A	0.9800
Cu1—N2	1.978 (2)	C4—H4B	0.9800
Cu1—N3	2.058 (2)	C4—H4C	0.9800
N1—C2	1.496 (4)	C5—H5	0.9500
N1—C3	1.482 (4)	C5—C6	1.460 (4)
N1—C4	1.482 (4)	C6—C7	1.380 (4)
N2—C1	1.469 (4)	C7—H7	0.9500
N2—C5	1.274 (4)	C7—C8	1.384 (5)
N3—C6	1.357 (4)	C8—H8	0.9500
N3—C10	1.328 (4)	C8—C9	1.383 (5)
C1—H1A	0.9900	C9—H9	0.9500
C1—H1B	0.9900	C9—C10	1.390 (4)
C1—C2	1.530 (4)	C10—H10	0.9500
C2—H2A	0.9900	O1—H1D	0.838 (10)
C2—H2B	0.9900	O1—H1C	0.837 (10)
C3—H3A	0.9800		
			
Cl2—Cu1—Cl1	102.95 (3)	H2A—C2—H2B	108.1
N1—Cu1—Cl1	99.55 (7)	N1—C3—H3A	109.5
N1—Cu1—Cl2	96.54 (7)	N1—C3—H3B	109.5
N2—Cu1—Cl1	97.12 (7)	N1—C3—H3C	109.5
N2—Cu1—Cl2	159.89 (8)	H3A—C3—H3B	109.5
N2—Cu1—N1	81.17 (10)	H3A—C3—H3C	109.5
N2—Cu1—N3	79.40 (10)	H3B—C3—H3C	109.5
N3—Cu1—Cl1	96.13 (7)	N1—C4—H4A	109.5
N3—Cu1—Cl2	97.08 (7)	N1—C4—H4B	109.5
N3—Cu1—N1	156.35 (10)	N1—C4—H4C	109.5
C2—N1—Cu1	105.54 (17)	H4A—C4—H4B	109.5
C3—N1—Cu1	107.23 (18)	H4A—C4—H4C	109.5
C3—N1—C2	110.9 (2)	H4B—C4—H4C	109.5
C4—N1—Cu1	115.69 (18)	N2—C5—H5	122.2
C4—N1—C2	108.9 (2)	N2—C5—C6	115.6 (3)
C4—N1—C3	108.6 (2)	C6—C5—H5	122.2
C1—N2—Cu1	117.93 (18)	N3—C6—C5	114.7 (2)
C5—N2—Cu1	117.8 (2)	N3—C6—C7	121.9 (3)
C5—N2—C1	124.2 (3)	C7—C6—C5	123.4 (3)
C6—N3—Cu1	112.31 (19)	C6—C7—H7	120.4
C10—N3—Cu1	129.0 (2)	C6—C7—C8	119.1 (3)
C10—N3—C6	118.7 (2)	C8—C7—H7	120.4
N2—C1—H1A	110.5	C7—C8—H8	120.5
N2—C1—H1B	110.5	C9—C8—C7	119.0 (3)
N2—C1—C2	106.0 (2)	C9—C8—H8	120.5
H1A—C1—H1B	108.7	C8—C9—H9	120.6
C2—C1—H1A	110.5	C8—C9—C10	118.8 (3)
C2—C1—H1B	110.5	C10—C9—H9	120.6
N1—C2—C1	110.2 (2)	N3—C10—C9	122.5 (3)
N1—C2—H2A	109.6	N3—C10—H10	118.7
N1—C2—H2B	109.6	C9—C10—H10	118.7
C1—C2—H2A	109.6	H1D—O1—H1C	102 (2)
C1—C2—H2B	109.6		
			
Cu1—N1—C2—C1	47.7 (3)	C3—N1—C2—C1	−68.1 (3)
Cu1—N2—C1—C2	12.6 (3)	C4—N1—C2—C1	172.5 (2)
Cu1—N2—C5—C6	4.5 (3)	C5—N2—C1—C2	−165.9 (3)
Cu1—N3—C6—C5	−1.8 (3)	C5—C6—C7—C8	−178.8 (3)
Cu1—N3—C6—C7	179.9 (2)	C6—N3—C10—C9	0.8 (4)
Cu1—N3—C10—C9	−179.2 (2)	C6—C7—C8—C9	0.6 (4)
N2—C1—C2—N1	−39.5 (3)	C7—C8—C9—C10	0.0 (4)
N2—C5—C6—N3	−1.6 (4)	C8—C9—C10—N3	−0.8 (4)
N2—C5—C6—C7	176.7 (3)	C10—N3—C6—C5	178.2 (2)
N3—C6—C7—C8	−0.6 (4)	C10—N3—C6—C7	−0.1 (4)
C1—N2—C5—C6	−176.9 (3)		

### Dichlorido[N,N-dimethyl-N'-(pyridin-2-ylmethylidene)ethane-1,2-diamine]copper(II) monohydrate (1) Hydrogen-bond geometry (Å, º)

**Table:**

*D*—H···*A*	*D*—H	H···*A*	*D*···*A*	*D*—H···*A*
O1—H1*D*···Cl1^i^	0.84 (1)	2.45 (1)	3.282 (3)	173 (5)
O1—H1*C*···Cl1^ii^	0.84 (1)	2.43 (1)	3.252 (3)	166 (4)
C5—H5···Cl1^iii^	0.95	2.73	3.669 (3)	171
C9—H9···Cl2^iv^	0.95	2.86	3.543 (3)	130

Symmetry codes: (i) *x*+1/2, −*y*+1/2, *z*+1/2; (ii) *x*+1, *y*, *z*+1; (iii) *x*−1, *y*, *z*; (iv) −*x*+1, −*y*, −*z*.

### µ2-Chlorido-dichlorido(µ2-2-{[2-(dimethylamino)ethyl]iminomethyl}phenolato)(N,N-dimethylethylenediamine)dicopper(II) 0.11-hydrate (2) Crystal data

**Table:**

[Cu_2_(C_11_H_15_N_2_O)Cl_3_(C_4_H_12_N_2_)]·0.11H_2_O	*F*(000) = 1051
*M**_r_* = 514.77	*D*_x_ = 1.631 Mg m^−^^3^
Monoclinic, *P*2_1_/*c*	Cu *K*α radiation, λ = 1.54178 Å
*a* = 11.0838 (4) Å	Cell parameters from 6733 reflections
*b* = 18.0949 (7) Å	θ = 4.8–68.2°
*c* = 10.6610 (4) Å	µ = 6.12 mm^−^^1^
β = 101.474 (2)°	*T* = 150 K
*V* = 2095.44 (14) Å^3^	Needle, clear green
*Z* = 4	0.08 × 0.06 × 0.04 mm

### µ2-Chlorido-dichlorido(µ2-2-{[2-(dimethylamino)ethyl]iminomethyl}phenolato)(N,N-dimethylethylenediamine)dicopper(II) 0.11-hydrate (2) Data collection

**Table:**

Bruker APEXII CCD area-detector diffractometer	3333 reflections with *I* > 2σ(*I*)
φ and ω scans	*R*_int_ = 0.024
Absorption correction: multi-scan (*SADABS*; Bruker, 2010)	θ_max_ = 68.2°, θ_min_ = 4.1°
*T*_min_ = 0.654, *T*_max_ = 0.753	*h* = −11→13
11774 measured reflections	*k* = −19→21
3680 independent reflections	*l* = −11→12

### µ2-Chlorido-dichlorido(µ2-2-{[2-(dimethylamino)ethyl]iminomethyl}phenolato)(N,N-dimethylethylenediamine)dicopper(II) 0.11-hydrate (2) Refinement

**Table:**

Refinement on *F*^2^	0 restraints
Least-squares matrix: full	Hydrogen site location: mixed
*R*[*F*^2^ > 2σ(*F*^2^)] = 0.022	H atoms treated by a mixture of independent and constrained refinement
*wR*(*F*^2^) = 0.056	*w* = 1/[σ^2^(*F*_o_^2^) + (0.0287*P*)^2^ + 1.0606*P*] where *P* = (*F*_o_^2^ + 2*F*_c_^2^)/3
*S* = 1.03	(Δ/σ)_max_ = 0.001
3680 reflections	Δρ_max_ = 0.32 e Å^−^^3^
248 parameters	Δρ_min_ = −0.28 e Å^−^^3^

### µ2-Chlorido-dichlorido(µ2-2-{[2-(dimethylamino)ethyl]iminomethyl}phenolato)(N,N-dimethylethylenediamine)dicopper(II) 0.11-hydrate (2) Special details

**Table:**

Geometry. All esds (except the esd in the dihedral angle between two l.s. planes) are estimated using the full covariance matrix. The cell esds are taken into account individually in the estimation of esds in distances, angles and torsion angles; correlations between esds in cell parameters are only used when they are defined by crystal symmetry. An approximate (isotropic) treatment of cell esds is used for estimating esds involving l.s. planes.

### µ2-Chlorido-dichlorido(µ2-2-{[2-(dimethylamino)ethyl]iminomethyl}phenolato)(N,N-dimethylethylenediamine)dicopper(II) 0.11-hydrate (2) Fractional atomic coordinates and isotropic or equivalent isotropic displacement parameters (Å^2^)

**Table:**

	*x*	*y*	*z*	*U*_iso_*/*U*_eq_	Occ. (<1)
Cu1	0.79793 (2)	0.56169 (2)	0.73980 (2)	0.01442 (8)	
Cu2	0.69862 (2)	0.39361 (2)	0.68114 (2)	0.01267 (8)	
Cl1	0.59567 (4)	0.62588 (3)	0.64539 (4)	0.02241 (11)	
Cl2	0.75397 (4)	0.47616 (3)	0.88333 (4)	0.02295 (11)	
Cl3	0.85942 (4)	0.32302 (2)	0.65652 (4)	0.02100 (11)	
O1	0.76725 (11)	0.48317 (7)	0.60963 (11)	0.0151 (3)	
N1	0.85544 (14)	0.64035 (9)	0.87958 (15)	0.0189 (3)	
N2	0.90614 (13)	0.61412 (8)	0.64520 (14)	0.0161 (3)	
N3	0.52655 (14)	0.43584 (9)	0.63814 (16)	0.0165 (3)	
H3A	0.503 (2)	0.4342 (12)	0.556 (2)	0.017 (5)*	
H3B	0.533 (2)	0.4801 (15)	0.663 (2)	0.024 (6)*	
N4	0.61771 (14)	0.31151 (8)	0.76637 (14)	0.0157 (3)	
C1	0.96901 (18)	0.67789 (11)	0.71453 (19)	0.0213 (4)	
H1A	0.9791	0.7178	0.6539	0.026*	
H1B	1.0515	0.6634	0.7625	0.026*	
C2	0.88892 (18)	0.70405 (11)	0.80598 (19)	0.0219 (4)	
H2A	0.9341	0.7413	0.8655	0.026*	
H2B	0.8133	0.7275	0.7571	0.026*	
C3	0.75991 (19)	0.66273 (12)	0.9508 (2)	0.0273 (5)	
H3C	0.6865	0.6794	0.8904	0.041*	
H3D	0.7913	0.7031	1.0096	0.041*	
H3E	0.7385	0.6205	0.9997	0.041*	
C4	0.96456 (18)	0.61355 (12)	0.97277 (19)	0.0262 (4)	
H4A	0.9411	0.5710	1.0195	0.039*	
H4B	0.9955	0.6532	1.0333	0.039*	
H4C	1.0291	0.5988	0.9270	0.039*	
C5	0.92226 (16)	0.59984 (10)	0.53223 (17)	0.0161 (4)	
H5	0.9786	0.6302	0.4993	0.019*	
C6	0.86234 (15)	0.54160 (10)	0.45045 (17)	0.0140 (3)	
C7	0.88041 (17)	0.54119 (11)	0.32361 (18)	0.0182 (4)	
H7	0.9342	0.5766	0.2983	0.022*	
C8	0.82217 (18)	0.49076 (11)	0.23504 (17)	0.0206 (4)	
H8	0.8345	0.4916	0.1494	0.025*	
C9	0.74469 (18)	0.43840 (11)	0.27382 (18)	0.0203 (4)	
H9	0.7030	0.4038	0.2134	0.024*	
C10	0.72765 (17)	0.43609 (10)	0.39835 (18)	0.0173 (4)	
H10	0.6757	0.3992	0.4226	0.021*	
C11	0.78550 (15)	0.48712 (10)	0.48998 (16)	0.0132 (3)	
C12	0.43951 (17)	0.39331 (11)	0.6993 (2)	0.0232 (4)	
H12A	0.3886	0.3606	0.6354	0.028*	
H12B	0.3839	0.4276	0.7329	0.028*	
C13	0.51174 (18)	0.34742 (11)	0.80794 (19)	0.0215 (4)	
H13A	0.5419	0.3795	0.8828	0.026*	
H13B	0.4575	0.3092	0.8337	0.026*	
C14	0.57543 (19)	0.25295 (11)	0.67056 (19)	0.0229 (4)	
H14A	0.5221	0.2747	0.5953	0.034*	
H14B	0.5294	0.2155	0.7080	0.034*	
H14C	0.6469	0.2299	0.6451	0.034*	
C15	0.69955 (18)	0.27786 (12)	0.87843 (19)	0.0240 (4)	
H15A	0.7701	0.2548	0.8512	0.036*	
H15B	0.6539	0.2403	0.9162	0.036*	
H15C	0.7288	0.3162	0.9421	0.036*	
O1W	0.4947 (19)	0.4479 (16)	0.081 (2)	0.084 (11)	0.108 (8)

### µ2-Chlorido-dichlorido(µ2-2-{[2-(dimethylamino)ethyl]iminomethyl}phenolato)(N,N-dimethylethylenediamine)dicopper(II) 0.11-hydrate (2) Atomic displacement parameters (Å^2^)

**Table:**

	*U*^11^	*U*^22^	*U*^33^	*U*^12^	*U*^13^	*U*^23^
Cu1	0.01696 (14)	0.01273 (14)	0.01364 (13)	−0.00351 (10)	0.00323 (10)	−0.00212 (10)
Cu2	0.01273 (13)	0.01071 (14)	0.01505 (13)	−0.00016 (10)	0.00394 (10)	0.00254 (10)
Cl1	0.0169 (2)	0.0237 (2)	0.0246 (2)	0.00368 (18)	−0.00074 (17)	−0.00630 (19)
Cl2	0.0342 (3)	0.0210 (2)	0.0129 (2)	−0.00864 (19)	0.00281 (17)	0.00016 (17)
Cl3	0.0164 (2)	0.0176 (2)	0.0312 (2)	0.00427 (17)	0.01032 (18)	0.00640 (18)
O1	0.0210 (6)	0.0116 (6)	0.0139 (6)	−0.0022 (5)	0.0063 (5)	0.0001 (5)
N1	0.0182 (8)	0.0187 (8)	0.0193 (8)	−0.0027 (6)	0.0027 (6)	−0.0053 (6)
N2	0.0148 (7)	0.0136 (7)	0.0200 (8)	−0.0019 (6)	0.0032 (6)	−0.0018 (6)
N3	0.0167 (8)	0.0148 (8)	0.0174 (8)	0.0000 (6)	0.0018 (6)	0.0006 (7)
N4	0.0170 (7)	0.0149 (7)	0.0163 (7)	0.0014 (6)	0.0062 (6)	0.0041 (6)
C1	0.0219 (9)	0.0170 (9)	0.0254 (10)	−0.0089 (8)	0.0059 (8)	−0.0049 (8)
C2	0.0234 (9)	0.0145 (9)	0.0275 (10)	−0.0032 (8)	0.0046 (8)	−0.0054 (8)
C3	0.0266 (10)	0.0310 (11)	0.0258 (10)	−0.0024 (9)	0.0087 (8)	−0.0123 (9)
C4	0.0241 (10)	0.0290 (11)	0.0227 (10)	−0.0022 (9)	−0.0024 (8)	−0.0021 (9)
C5	0.0132 (8)	0.0149 (9)	0.0206 (9)	−0.0008 (7)	0.0046 (7)	0.0033 (7)
C6	0.0120 (8)	0.0132 (8)	0.0168 (8)	0.0015 (7)	0.0028 (7)	0.0014 (7)
C7	0.0172 (9)	0.0187 (9)	0.0201 (9)	0.0005 (8)	0.0074 (7)	0.0026 (8)
C8	0.0251 (9)	0.0226 (10)	0.0155 (9)	0.0024 (8)	0.0070 (7)	−0.0003 (8)
C9	0.0241 (10)	0.0180 (10)	0.0179 (9)	0.0009 (8)	0.0018 (7)	−0.0032 (7)
C10	0.0191 (9)	0.0133 (9)	0.0195 (9)	−0.0018 (7)	0.0038 (7)	−0.0005 (7)
C11	0.0131 (8)	0.0114 (8)	0.0153 (8)	0.0036 (7)	0.0034 (6)	0.0020 (7)
C12	0.0162 (9)	0.0239 (10)	0.0312 (10)	0.0017 (8)	0.0087 (8)	0.0043 (8)
C13	0.0206 (9)	0.0229 (10)	0.0238 (10)	0.0015 (8)	0.0114 (8)	0.0025 (8)
C14	0.0267 (10)	0.0170 (9)	0.0264 (10)	−0.0066 (8)	0.0086 (8)	0.0000 (8)
C15	0.0240 (10)	0.0247 (10)	0.0240 (10)	0.0030 (8)	0.0067 (8)	0.0105 (8)
O1W	0.048 (13)	0.12 (2)	0.081 (17)	0.002 (13)	0.006 (11)	−0.024 (15)

### µ2-Chlorido-dichlorido(µ2-2-{[2-(dimethylamino)ethyl]iminomethyl}phenolato)(N,N-dimethylethylenediamine)dicopper(II) 0.11-hydrate (2) Geometric parameters (Å, º)

**Table:**

Cu1—Cl1	2.5476 (5)	C3—H3D	0.9800
Cu1—Cl2	2.2957 (5)	C3—H3E	0.9800
Cu1—O1	1.9679 (12)	C4—H4A	0.9800
Cu1—N1	2.0675 (15)	C4—H4B	0.9800
Cu1—N2	1.9589 (15)	C4—H4C	0.9800
Cu2—Cl2	2.5939 (5)	C5—H5	0.9500
Cu2—Cl3	2.2500 (5)	C5—C6	1.443 (3)
Cu2—O1	2.0042 (12)	C6—C7	1.406 (3)
Cu2—N3	2.0209 (16)	C6—C11	1.420 (2)
Cu2—N4	2.0399 (15)	C7—H7	0.9500
O1—C11	1.333 (2)	C7—C8	1.378 (3)
N1—C2	1.483 (3)	C8—H8	0.9500
N1—C3	1.477 (2)	C8—C9	1.395 (3)
N1—C4	1.486 (2)	C9—H9	0.9500
N2—C1	1.469 (2)	C9—C10	1.378 (3)
N2—C5	1.279 (2)	C10—H10	0.9500
N3—H3A	0.87 (2)	C10—C11	1.403 (3)
N3—H3B	0.84 (3)	C12—H12A	0.9900
N3—C12	1.483 (2)	C12—H12B	0.9900
N4—C13	1.485 (2)	C12—C13	1.517 (3)
N4—C14	1.482 (3)	C13—H13A	0.9900
N4—C15	1.480 (2)	C13—H13B	0.9900
C1—H1A	0.9900	C14—H14A	0.9800
C1—H1B	0.9900	C14—H14B	0.9800
C1—C2	1.519 (3)	C14—H14C	0.9800
C2—H2A	0.9900	C15—H15A	0.9800
C2—H2B	0.9900	C15—H15B	0.9800
C3—H3C	0.9800	C15—H15C	0.9800
			
Cl2—Cu1—Cl1	106.496 (19)	N1—C3—H3C	109.5
O1—Cu1—Cl1	92.04 (4)	N1—C3—H3D	109.5
O1—Cu1—Cl2	87.33 (4)	N1—C3—H3E	109.5
O1—Cu1—N1	172.12 (6)	H3C—C3—H3D	109.5
N1—Cu1—Cl1	95.27 (5)	H3C—C3—H3E	109.5
N1—Cu1—Cl2	93.42 (5)	H3D—C3—H3E	109.5
N2—Cu1—Cl1	99.01 (5)	N1—C4—H4A	109.5
N2—Cu1—Cl2	154.49 (5)	N1—C4—H4B	109.5
N2—Cu1—O1	91.39 (6)	N1—C4—H4C	109.5
N2—Cu1—N1	84.55 (6)	H4A—C4—H4B	109.5
Cl3—Cu2—Cl2	111.221 (19)	H4A—C4—H4C	109.5
O1—Cu2—Cl2	78.78 (4)	H4B—C4—H4C	109.5
O1—Cu2—Cl3	92.60 (4)	N2—C5—H5	117.2
O1—Cu2—N3	91.17 (6)	N2—C5—C6	125.67 (17)
O1—Cu2—N4	172.78 (6)	C6—C5—H5	117.2
N3—Cu2—Cl2	91.56 (5)	C7—C6—C5	116.65 (16)
N3—Cu2—Cl3	157.21 (5)	C7—C6—C11	119.36 (16)
N3—Cu2—N4	84.13 (6)	C11—C6—C5	123.97 (16)
N4—Cu2—Cl2	95.82 (4)	C6—C7—H7	119.1
N4—Cu2—Cl3	93.85 (4)	C8—C7—C6	121.71 (17)
Cu1—Cl2—Cu2	83.156 (16)	C8—C7—H7	119.1
Cu1—O1—Cu2	109.94 (6)	C7—C8—H8	120.7
C11—O1—Cu1	126.76 (11)	C7—C8—C9	118.59 (17)
C11—O1—Cu2	123.29 (11)	C9—C8—H8	120.7
C2—N1—Cu1	103.16 (11)	C8—C9—H9	119.5
C2—N1—C4	110.58 (15)	C10—C9—C8	121.09 (18)
C3—N1—Cu1	114.17 (12)	C10—C9—H9	119.5
C3—N1—C2	109.84 (16)	C9—C10—H10	119.4
C3—N1—C4	108.49 (15)	C9—C10—C11	121.29 (17)
C4—N1—Cu1	110.53 (12)	C11—C10—H10	119.4
C1—N2—Cu1	113.52 (12)	O1—C11—C6	122.37 (16)
C5—N2—Cu1	126.99 (13)	O1—C11—C10	119.72 (16)
C5—N2—C1	119.44 (16)	C10—C11—C6	117.91 (16)
Cu2—N3—H3A	107.4 (14)	N3—C12—H12A	109.8
Cu2—N3—H3B	105.4 (16)	N3—C12—H12B	109.8
H3A—N3—H3B	110 (2)	N3—C12—C13	109.21 (15)
C12—N3—Cu2	111.79 (12)	H12A—C12—H12B	108.3
C12—N3—H3A	109.6 (14)	C13—C12—H12A	109.8
C12—N3—H3B	112.5 (16)	C13—C12—H12B	109.8
C13—N4—Cu2	104.96 (11)	N4—C13—C12	109.90 (15)
C14—N4—Cu2	108.70 (11)	N4—C13—H13A	109.7
C14—N4—C13	110.99 (15)	N4—C13—H13B	109.7
C15—N4—Cu2	113.96 (11)	C12—C13—H13A	109.7
C15—N4—C13	109.47 (14)	C12—C13—H13B	109.7
C15—N4—C14	108.74 (15)	H13A—C13—H13B	108.2
N2—C1—H1A	110.4	N4—C14—H14A	109.5
N2—C1—H1B	110.4	N4—C14—H14B	109.5
N2—C1—C2	106.76 (15)	N4—C14—H14C	109.5
H1A—C1—H1B	108.6	H14A—C14—H14B	109.5
C2—C1—H1A	110.4	H14A—C14—H14C	109.5
C2—C1—H1B	110.4	H14B—C14—H14C	109.5
N1—C2—C1	109.70 (16)	N4—C15—H15A	109.5
N1—C2—H2A	109.7	N4—C15—H15B	109.5
N1—C2—H2B	109.7	N4—C15—H15C	109.5
C1—C2—H2A	109.7	H15A—C15—H15B	109.5
C1—C2—H2B	109.7	H15A—C15—H15C	109.5
H2A—C2—H2B	108.2	H15B—C15—H15C	109.5

### µ2-Chlorido-dichlorido(µ2-2-{[2-(dimethylamino)ethyl]iminomethyl}phenolato)(N,N-dimethylethylenediamine)dicopper(II) 0.11-hydrate (2) Hydrogen-bond geometry (Å, º)

**Table:**

*D*—H···*A*	*D*—H	H···*A*	*D*···*A*	*D*—H···*A*
N3—H3*A*···Cl1^i^	0.87 (2)	2.46 (2)	3.2543 (17)	153 (2)
N3—H3*B*···Cl1	0.84 (2)	2.74 (2)	3.5207 (1)	154 (2)
C8—H8···Cl2^ii^	0.95	2.81	3.6841 (19)	153
O1*W*···Cl2^i^			3.17 (2)	

Symmetry codes: (i) −*x*+1, −*y*+1, −*z*+1; (ii) *x*, *y*, *z*−1.
